# Retraction: The *Plasmodium* circumsporozoite protein, a novel NF-κB inhibitor, suppresses the growth of SW480

**DOI:** 10.3389/pore.2024.1612018

**Published:** 2024-11-12

**Authors:** Pathology and Oncology Research Editorial Office

Following publication, concerns were raised on the PubPeer platform regarding the integrity of the images in the published figures, in particular potential image duplication was identified in Figure 2A. The authors of the manuscript then contacted the POR Editorial Office to request a correction to the figure in question.

**FIGURE 2:**
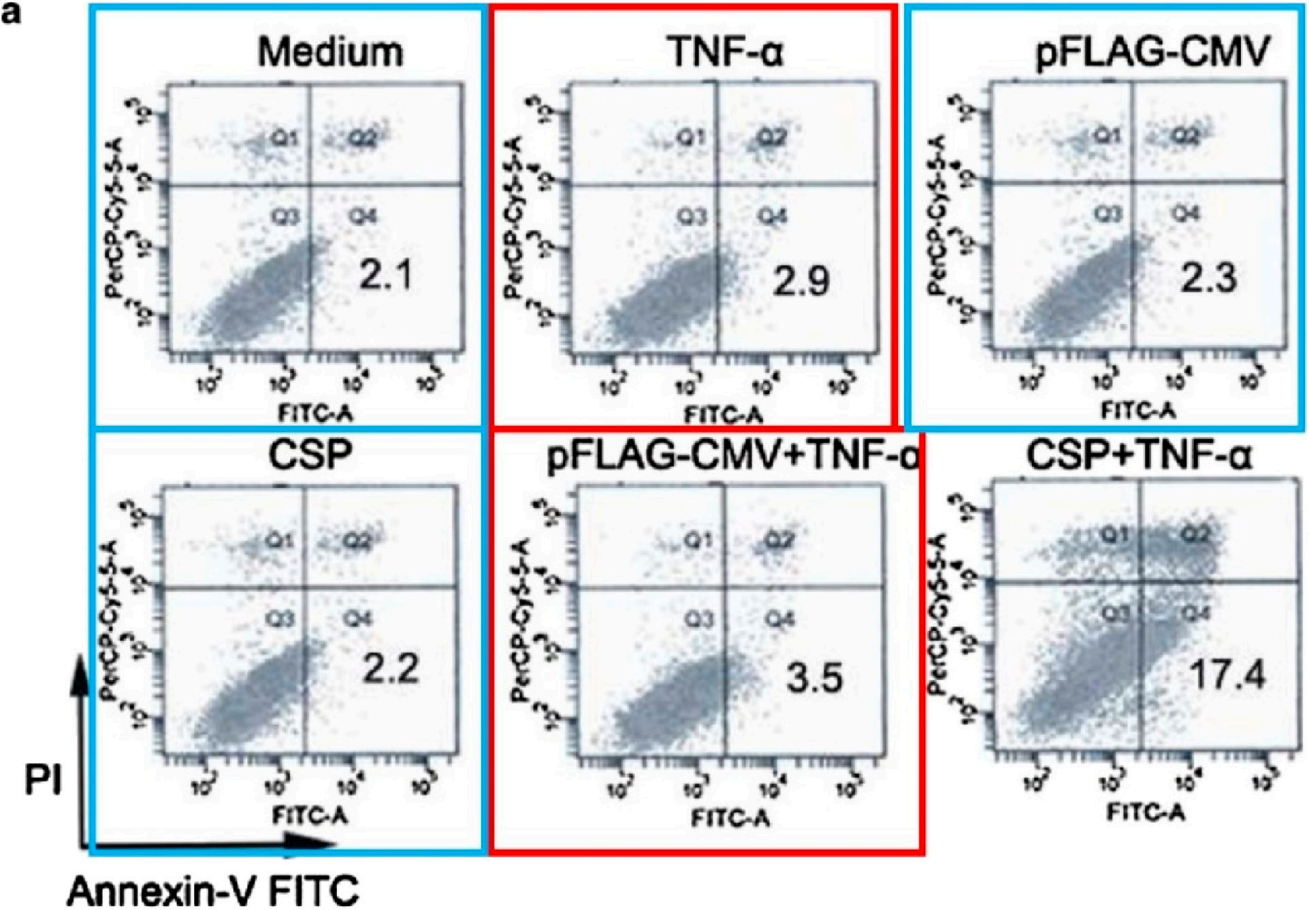
The effects of the CSP on the apoptosis of SW480. **(A)** After transfection of SW480 with or without 0.8 μg pFLAG-CMV8 or pFLAG-CMV8-CSP for 24 h, the cells were cultured alone or stimulated with 100 ng/mL hTNF-α for 6 h. Next, the cells were harvested and labeled with Annexin V-FITC and PI and analyzed using flow cytometry. **(B)** The percentage of apoptotic SW480 following transfection with or without 0.8 μg pFLAG-CMV8 or pFLAG-CMV8-CSP followed hTNF-α stimulation were compared. The experiments were performed in triplicate, and the data are expressed as the M±SD. (**p < 0.01).

Following provision of raw data by the authors, the Editor-in-Chief concluded that the article’s conclusions and assertions were not sufficiently supported by the findings from the material provided; therefore, the article has been retracted.

This retraction was approved by the Editor-in-Chief of POR. The authors received a communication regarding the retraction and had a chance to respond. This communication has been recorded by the publisher.

